# “Black” and “White” Blood on Unenhanced CT

**DOI:** 10.5334/jbsr.1733

**Published:** 2019-02-04

**Authors:** Olivia del Marmol, Bruno Coulier

**Affiliations:** 1 Clinique Saint-Luc, Bouge, BE

**Keywords:** Blood density, anemia, unenhanced CT, hypodensity, hyperdensity

## Case Report

**Figure 1:**
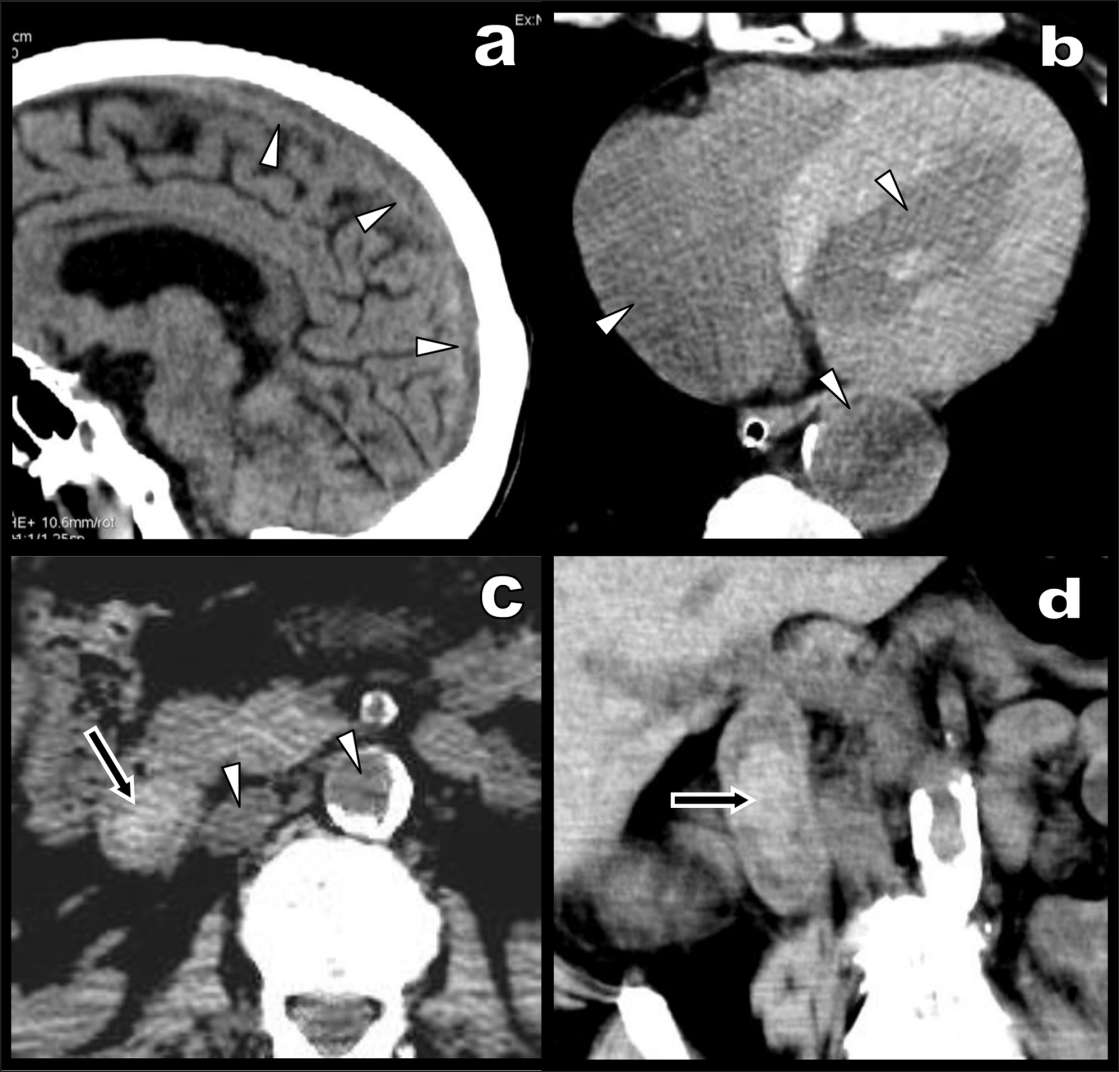
Unenhanced CT views: **(a)** Sagital MPR view obtained at the level of the longitudinal venous sinus of the brain shows hypodensity of blood (white arrowheads); **(b)** marked hypodensity of blood (25–30 UH) is also found in the cardiac cavities (white arrowheads); axial **(c)** and coronal MPR views **(d)** reveal a spontaneously dense fresh clot in the 2^nd^ duodenum (black arrow). Its high density (60 UH) contrasts with the high hypodensity of blood (25–30 UH) in the great abdominal vessels (white arrowheads).

A 70-year-old man was admitted unconscious. Despite recent recurrent episodes of melena and alteration of his general condition, the patient had stubbornly refused any hospitalization. Pallor, hypothermia, severe hypotension and bradycardia were noticed at arrival. Unenhanced emergency brain and body computed tomography (CT) were performed (Figure 1). Spontaneous hypodensity of blood comprised between 25–30 Hounsfield units (HU) was diffusely found in cerebral venous sinuses (a, white arrowheads on sagittal view), in the body large vessels (c, white arrowheads in the abdominal aorta and vena cava) and the cardiac cavities (b, white arrowheads). This hypodensity contrasted markedly with the spontaneous luminal hyperdensity (60 HU) in the second duodenum (black arrow on axial [c] and coronal [d] views). The preliminary diagnosis of severe anemia resulting from recent bleeding in the upper gastrointestinal tract was proposed.

Laboratory tests confirmed hemoglobin concentration at 57 g/l and 18.4% hematocrit. Emergency gastroscopy found active bleeding from gastroduodenal ulcerations.

## Comment

This article emphasizes the importance of “black” or “white” blood on unenhanced CT. Radiologists are trained to specifically recognize hyper attenuating vascular signs. Blood pool hypodensity is more unusual and may be subtle. The simultaneous association of hypo- and hyperdensity of blood in the same patient is uncommon but important for the diagnosis. The CT attenuation of in vitro blood primarily relates to the concentration of erythrocytes and the protein fraction of hemoglobin, which has a high-density mass; the contribution of hemoglobin iron atom itself is minimal. There is a linear relationship between hematocrit and CT attenuation of blood [1]. Thus, normal flowing blood has a density about 40 HU. Attenuation measures of <35 HU in the superior sagittal sinus or the left ventricle are highly predictive of anemia. Reciprocally, others have shown that an aortic attenuation value in excess of 50 HU males and 45 HU in a females denote absence of anemia. Conversely, when a blood clot retracts, its water content decreases and its hematocrit may raise up to 90%. As a result, fresh blood clots typically appear hyperdense. Diffuse hyperdensity of all vessels also represents a rare phenomenon characteristically found in polycythemia when the hematocrit value exceeds 60%.
